# 1-Benzothio­phene-2-carbaldehyde 4-ethyl­thio­semicarbazone

**DOI:** 10.1107/S1600536808042797

**Published:** 2008-12-20

**Authors:** Safa’a Fares Kayed, Yang Farina, Jim Simpson

**Affiliations:** a School of Chemical Sciences and Food Technology, Faculty of Science and Technology, Universiti Kebangsaan Malaysia, 43600 UKM Bangi, Selangor, Malaysia; b Department of Chemistry, University of Otago, PO Box 56, Dunedin, New Zealand

## Abstract

The title compound, C_13_H_15_N_3_S_2_, crystallizes with two unique mol­ecules, *A* and *B*, in the asymmetric unit. These differ principally in that the methyl group of the 4-ethyl­thio­semicarbazone moiety is ordered in mol­ecule *A* but disordered over two positions with equal occupancies in mol­ecule *B*. The benzothio­phene group and the semicarbazone unit are inclined at dihedral angles of 11.78 (8)° for mol­ecule *A* and 8.18 (13)° for mol­ecule *B*. Weak intra­molecular N—H⋯N inter­actions contribute to the planarity of the semicarbazone units in both mol­ecules and each mol­ecule adopts an *E* configuration with respect to the C=N bonds. In the crystal structure, mol­ecules form centrosymmetric dimers as a result of N—H⋯S hydrogen bonds, augmented by C—H⋯S inter­actions for mol­ecule *A* and C—H⋯S inter­actions for mol­ecule *B*. Weak C—H⋯π inter­actions stack the dimers of both mol­ecules into columns down the *a* axis.

## Related literature

For background to the biological activity of thio­semicarbazones, see: de Sousa *et al.* (2007 ▶). For related structures, see: Chuev *et al.* (1992 ▶); de Lima *et al.* (2002 ▶); Isik *et al.* (2006 ▶); Kayed *et al.* (2008 ▶). For details of graph-set analysis of hydrogen-bonding patterns, see: Bernstein *et al.* (1995 ▶). For reference structural data, see: Allen *et al.* (1987 ▶).
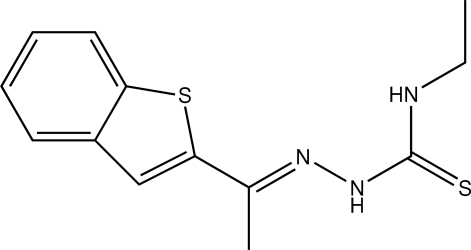


## Experimental

### 

#### Crystal data

C_13_H_15_N_3_S_2_
*M* *_r_* = 277.40
Triclinic, 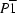

*a* = 5.5343 (5) Å
*b* = 10.9943 (10) Å
*c* = 23.443 (2) Å
α = 78.825 (5)°
β = 88.175 (5)°
γ = 76.298 (5)°
*V* = 1359.4 (2) Å^3^
*Z* = 4
Mo *K*α radiation
μ = 0.38 mm^−1^
*T* = 92 (2) K
0.37 × 0.10 × 0.05 mm

#### Data collection

Bruker APEXII CCD area-detector diffractometer
Absorption correction: multi-scan (*SADABS*; Bruker, 2006 ▶) *T*_min_ = 0.873, *T*_max_ = 0.981
18044 measured reflections
5907 independent reflections
4307 reflections with *I* > 2σ(*I*)
*R*_int_ = 0.051

#### Refinement

*R*[*F* ^2^ > 2σ(*F* ^2^)] = 0.042
*wR*(*F* ^2^) = 0.111
*S* = 1.05
5907 reflections
354 parameters
6 restraints
H atoms treated by a mixture of independent and constrained refinement
Δρ_max_ = 0.49 e Å^−3^
Δρ_min_ = −0.41 e Å^−3^

### 

Data collection: *APEX2* (Bruker 2006 ▶); cell refinement: *APEX2* and *SAINT* (Bruker 2006 ▶); data reduction: *SAINT*; program(s) used to solve structure: *SHELXS97* (Sheldrick, 2008 ▶); program(s) used to refine structure: *SHELXL97* (Sheldrick, 2008 ▶) and *TITAN* (Hunter & Simpson, 1999 ▶); molecular graphics: *SHELXTL* (Sheldrick, 2008 ▶) and *Mercury* (Macrae *et al.*, 2006 ▶); software used to prepare material for publication: *SHELXL97*, *enCIFer* (Allen *et al.*, 2004 ▶), *PLATON* (Spek, 2003 ▶) and *publCIF* (Westrip, 2009 ▶).

## Supplementary Material

Crystal structure: contains datablocks global, I. DOI: 10.1107/S1600536808042797/ci2731sup1.cif

Structure factors: contains datablocks I. DOI: 10.1107/S1600536808042797/ci2731Isup2.hkl

Additional supplementary materials:  crystallographic information; 3D view; checkCIF report

## Figures and Tables

**Table 1:** Hydrogen-bond geometry (Å, °)

*D*—H⋯*A*	*D*—H	H⋯*A*	*D*⋯*A*	*D*—H⋯*A*
N3*A*—H3*NA*⋯N1*A*	0.84 (1)	2.27 (2)	2.623 (3)	106 (2)
N3*B*—H3*NB*⋯N1*B*	0.83 (1)	2.26 (2)	2.627 (3)	107 (2)
C10*A*—H10*A*⋯S2*A*^i^	0.98	2.84	3.374 (2)	115
N2*A*—H2*NA*⋯S2*A*^i^	0.85 (1)	2.81 (1)	3.638 (2)	164 (2)
C10*B*—H10*D*⋯S2*B*^ii^	0.98	2.82	3.373 (2)	117
C10*A*—H10*B*⋯*Cg*1^iii^	0.98	2.71	3.577 (2)	147
C10*B*—H10*E*⋯*Cg*2^iv^	0.98	2.72	3.600 (2)	150

Symmetry codes: (i) 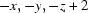; (ii) 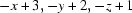; (iii) 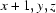; (iv) 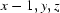. *Cg*1 and *Cg*2 are the centroids of the S1*A*/C1*A*/C2*A*/C3*A*/C8*A* and S1*B*/C1*B*/C2*B*/C3*B*/C8*B* rings, respectively.

**Fig. 1.**
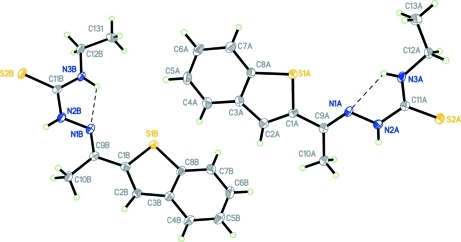
The asymmetric unit of the title compound, with atom labels and 50% probability displacement ellipsoids for non-H atoms. For clarity, only one disorder component is shown.

**Fig. 2.**
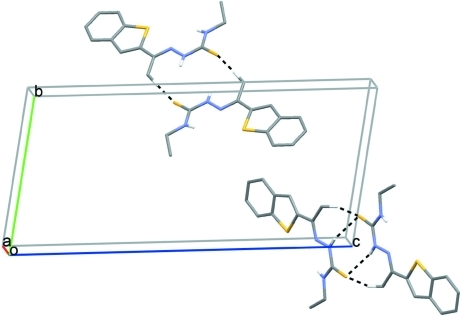
Formation of dimers in the crystal structure of the title compound, through N—H···S and C—H···S hydrogen bonds, shown as dashed lines. H atoms not involved in hydrogen bonding and atoms of one of the disorder components have been omitted for clarity.

**Fig. 3.**
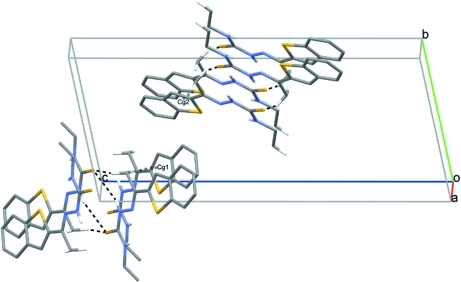
C—H···π interactions between dimers in the title compound. Cg1 is the centroid of the S1A/C1A/C2A/C3A/C8A ring and Cg2 that of the S1B/C1B/C2B/C3B/C8B ring. H···Cg interactions are drawn as dotted lines and hydrogen bonds as dashed lines. H atoms not involved in these interactions and atoms of one of the disorder components have been omitted for clarity.

## supplementary crystallographic information

### Comment

Thiosemicarbazones are a class of compounds that have been investigated because of their biological activity (de Sousa *et al.*, 2007). As a continuation of our work on thiosemicarbazone compounds as potential ligands in transition metal chemistry (Kayed *et al.*, 2008;) we report here the structure of the title compound (Fig. 1), which crystallizes with two unique molecules, A and B, in the asymmetric unit. The two molecules are closely similar with the exception of the methyl C atom of the ethyl group which is disordered over two positions C131 and C132, with equal occupancies. The similarities of the remainder of the two molecules are demonstrated by the fact that the non-hydrogen atoms of molecules A and B overlay in Mercury (Macrae *et al.*, 2006) with an r.m.s. deviation of 0.077 Å, when the C132 disorder component is excluded. The molecules are each reasonably planar with r.m.s. deviations of 0.137 Å and 0.128 Å from the planes through all non-hydrogen atoms of the two molecules excluding the C132 disorder component. The planarity of the N1/N2/C11/S2/N3 segments of both molecules (r.m.s. deviations 0.050 Å for molecule A and 0.037 Å for molecule B) is aided by weak intramolecular N3—H3N···N1 interactions. The benzothiophene groups and the semicarbazone groups are inclined at dihedral angles of 11.78 (8)° for molecule A and 8.18 (13)° for molecule B. Both molecules adopt an *E* configuration with respect to the C═N bonds, bond distances are normal (Allen *et al.*, 1987) and comparable to those in similar structures (Chuev *et al.* 1992; de Lima *et al.* 2002; Isik *et al.* 2006; Kayed *et al.* 2008).

In the crystal structure, a centrosymmetric dimer with an *R*^2^_2_(8) ring motif (Bernstein *et al.*, 1995) is formed by through N2A—H2NA···S2A hydrogen bonds strengthened by additional C10A—H10A···S2A interactions for molecule A. A second dimer forms *via* C10B—H10D···S2B interactions for molecule B (Table 1 and Fig. 2). The dimers are further aggregated into columns down the *a* axis by weak C—H···π interactions involving the C10A and C10B methyl groups and the thiophene rings of adjacent molecules, Fig. 3.

### Experimental

The title compound was prepared by heating 35 ml of an ethanolic solution of 2-acetylbenzothiophene (1.76 g, 10 mmol) and 4-ethyl-3-thiosemicarbazide (1.2 g, 10 mmol) under reflux for 2 h. Three drops of concentrated H_2_SO_4_ were added. The resulting product was isolated and recrystallized from acetonitrile to afford yellow needles of the title compound in 63.5% yield (m.p. 448–450 K).

### Refinement

H atoms bound to N2A, N2B, N3A and N3B were located in a difference electron density map and refined freely with *U*_iso_ = 1.2*U*_eq_(N). All other H-atoms were refined using a riding model with d(C-H) = 0.95 Å, *U*_iso_ = 1.2*U*_eq_ (C) for aromatic, and 0.98 Å and *U*_iso_ = 1.5*U*_eq_ (C) for CH_3_ H atoms. The methyl C atom of the ethyl group in molecule B is disordered over two positions, C131 and C132, each with occupancies of approximately 0.5. These occupancies were fixed at 0.5 in the final refinement cycles.

### Crystal data

**Table:**

C_13_H_15_N_3_S_2_	*Z* = 4
*M**_r_* = 277.40	*F*(000) = 584
Triclinic, *P*1	*D*_x_ = 1.355 Mg m^−^^3^
Hall symbol: -P 1	Mo *K*α radiation, λ = 0.71073 Å
*a* = 5.5343 (5) Å	Cell parameters from 3088 reflections
*b* = 10.9943 (10) Å	θ = 4.7–52.7°
*c* = 23.443 (2) Å	µ = 0.38 mm^−^^1^
α = 78.825 (5)°	*T* = 92 K
β = 88.175 (5)°	Needle, yellow
γ = 76.298 (5)°	0.37 × 0.10 × 0.05 mm
*V* = 1359.4 (2) Å^3^	

### Data collection

**Table:**

Bruker APEXII CCD area-detector diffractometer	5907 independent reflections
Radiation source: fine-focus sealed tube	4307 reflections with *I* > 2σ(*I*)
graphite	*R*_int_ = 0.051
ω scans	θ_max_ = 27.1°, θ_min_ = 0.9°
Absorption correction: multi-scan (*SADABS*; Bruker, 2006)	*h* = −7→6
*T*_min_ = 0.873, *T*_max_ = 0.981	*k* = −14→14
18044 measured reflections	*l* = −30→29

### Refinement

**Table:**

Refinement on *F*^2^	Primary atom site location: structure-invariant direct methods
Least-squares matrix: full	Secondary atom site location: difference Fourier map
*R*[*F*^2^ > 2σ(*F*^2^)] = 0.042	Hydrogen site location: inferred from neighbouring sites
*wR*(*F*^2^) = 0.111	H atoms treated by a mixture of independent and constrained refinement
*S* = 1.05	*w* = 1/[σ^2^(*F*_o_^2^) + (0.0519*P*)^2^] where *P* = (*F*_o_^2^ + 2*F*_c_^2^)/3
5907 reflections	(Δ/σ)_max_ = 0.001
354 parameters	Δρ_max_ = 0.49 e Å^−^^3^
6 restraints	Δρ_min_ = −0.41 e Å^−^^3^

### Special details

**Table:**

Geometry. All e.s.d.'s (except the e.s.d. in the dihedral angle between two l.s. planes) are estimated using the full covariance matrix. The cell e.s.d.'s are taken into account individually in the estimation of e.s.d.'s in distances, angles and torsion angles; correlations between e.s.d.'s in cell parameters are only used when they are defined by crystal symmetry. An approximate (isotropic) treatment of cell e.s.d.'s is used for estimating e.s.d.'s involving l.s. planes.
Refinement. Refinement of *F*^2^ against ALL reflections. The weighted *R*-factor *wR* and goodness of fit *S* are based on *F*^2^, conventional *R*-factors *R* are based on *F*, with *F* set to zero for negative *F*^2^. The threshold expression of *F*^2^ > σ(*F*^2^) is used only for calculating *R*-factors(gt) *etc*. and is not relevant to the choice of reflections for refinement. *R*-factors based on *F*^2^ are statistically about twice as large as those based on *F*, and *R*- factors based on ALL data will be even larger.

### Fractional atomic coordinates and isotropic or equivalent isotropic displacement parameters (Å^2^)

**Table:**

	*x*	*y*	*z*	*U*_iso_*/*U*_eq_	Occ. (<1)
S1A	0.93115 (11)	0.05062 (5)	0.82595 (2)	0.01751 (15)	
S2A	0.13864 (11)	−0.19901 (5)	0.99817 (3)	0.02041 (15)	
N1A	0.5271 (3)	0.01724 (17)	0.90359 (8)	0.0153 (4)	
N2A	0.3543 (3)	−0.02135 (17)	0.94186 (8)	0.0163 (4)	
H2NA	0.225 (3)	0.0337 (19)	0.9488 (10)	0.028 (7)*	
N3A	0.5742 (4)	−0.22703 (18)	0.94436 (8)	0.0170 (4)	
H3NA	0.691 (3)	−0.195 (2)	0.9300 (10)	0.022 (7)*	
C1A	0.6844 (4)	0.1718 (2)	0.84181 (9)	0.0150 (5)	
C2A	0.6914 (4)	0.2885 (2)	0.80979 (9)	0.0182 (5)	
H2A	0.5730	0.3648	0.8132	0.022*	
C3A	0.8956 (4)	0.2843 (2)	0.77044 (9)	0.0175 (5)	
C4A	0.9572 (5)	0.3831 (2)	0.72958 (10)	0.0238 (6)	
H4A	0.8591	0.4678	0.7260	0.029*	
C5A	1.1606 (5)	0.3570 (2)	0.69452 (10)	0.0253 (6)	
H5A	1.2001	0.4241	0.6665	0.030*	
C6A	1.3095 (5)	0.2332 (2)	0.69971 (10)	0.0241 (6)	
H6A	1.4497	0.2169	0.6755	0.029*	
C7A	1.2534 (4)	0.1345 (2)	0.73997 (10)	0.0222 (5)	
H7A	1.3555	0.0506	0.7440	0.027*	
C8A	1.0452 (4)	0.1596 (2)	0.77463 (9)	0.0162 (5)	
C9A	0.4991 (4)	0.1378 (2)	0.88361 (9)	0.0148 (5)	
C10A	0.2935 (4)	0.2411 (2)	0.89979 (10)	0.0203 (5)	
H10A	0.2988	0.2372	0.9419	0.030*	
H10B	0.1328	0.2289	0.8887	0.030*	
H10C	0.3146	0.3244	0.8793	0.030*	
C11A	0.3697 (4)	−0.1483 (2)	0.95961 (9)	0.0154 (5)	
C12A	0.6216 (4)	−0.3666 (2)	0.95906 (10)	0.0194 (5)	
H12A	0.4719	−0.3938	0.9496	0.023*	
H12B	0.6555	−0.3954	1.0013	0.023*	
C13A	0.8407 (4)	−0.4280 (2)	0.92596 (11)	0.0253 (6)	
H13A	0.8674	−0.5210	0.9362	0.038*	
H13B	0.9899	−0.4029	0.9361	0.038*	
H13C	0.8068	−0.4000	0.8841	0.038*	
S1B	0.67836 (11)	0.71828 (5)	0.64398 (3)	0.01964 (15)	
S2B	1.51802 (11)	0.85218 (6)	0.44850 (3)	0.02281 (16)	
N1B	1.0500 (3)	0.82960 (18)	0.57598 (8)	0.0180 (4)	
N2B	1.2186 (4)	0.87090 (19)	0.53790 (8)	0.0187 (4)	
H2NB	1.300 (4)	0.9225 (19)	0.5457 (11)	0.028 (8)*	
N3B	1.1624 (4)	0.72808 (19)	0.48311 (9)	0.0205 (5)	
H3NB	1.040 (3)	0.721 (2)	0.5042 (9)	0.022 (7)*	
C1B	0.8201 (4)	0.8337 (2)	0.66072 (10)	0.0159 (5)	
C2B	0.7499 (4)	0.8626 (2)	0.71398 (9)	0.0163 (5)	
H2B	0.8087	0.9234	0.7301	0.020*	
C3B	0.5797 (4)	0.7926 (2)	0.74310 (9)	0.0161 (5)	
C4B	0.4694 (4)	0.7975 (2)	0.79772 (10)	0.0207 (5)	
H4B	0.5074	0.8524	0.8210	0.025*	
C5B	0.3050 (4)	0.7218 (2)	0.81723 (11)	0.0241 (6)	
H5B	0.2285	0.7258	0.8539	0.029*	
C6B	0.2499 (4)	0.6392 (2)	0.78363 (11)	0.0256 (6)	
H6B	0.1380	0.5872	0.7979	0.031*	
C7B	0.3576 (4)	0.6328 (2)	0.72976 (11)	0.0234 (6)	
H7B	0.3197	0.5771	0.7069	0.028*	
C8B	0.5215 (4)	0.7090 (2)	0.70985 (10)	0.0179 (5)	
C9B	0.9933 (4)	0.8839 (2)	0.62066 (9)	0.0156 (5)	
C10B	1.0962 (4)	0.9905 (2)	0.63256 (10)	0.0204 (5)	
H10D	1.0649	1.0607	0.5988	0.031*	
H10E	1.2757	0.9602	0.6400	0.031*	
H10F	1.0154	1.0207	0.6667	0.031*	
C11B	1.2880 (4)	0.8130 (2)	0.49148 (9)	0.0163 (5)	
C12B	1.1924 (5)	0.6655 (2)	0.43327 (12)	0.0370 (7)	
H12C	1.3697	0.6441	0.4224	0.044*	0.50
H12D	1.0953	0.7219	0.3995	0.044*	0.50
H12E	1.2155	0.7286	0.4004	0.044*	0.50
H12F	1.0372	0.6450	0.4271	0.044*	0.50
C131	1.0968 (10)	0.5437 (4)	0.4516 (2)	0.0281 (12)	0.50
H13D	0.9176	0.5667	0.4586	0.042*	0.50
H13E	1.1834	0.4928	0.4873	0.042*	0.50
H13F	1.1282	0.4939	0.4205	0.042*	0.50
C132	1.3847 (8)	0.5534 (4)	0.4312 (2)	0.0265 (12)	0.50
H13G	1.5473	0.5719	0.4354	0.040*	0.50
H13H	1.3754	0.5271	0.3939	0.040*	0.50
H13I	1.3629	0.4846	0.4630	0.040*	0.50

### Atomic displacement parameters (Å^2^)

**Table:**

	*U*^11^	*U*^22^	*U*^33^	*U*^12^	*U*^13^	*U*^23^
S1A	0.0177 (3)	0.0162 (3)	0.0184 (3)	−0.0042 (2)	0.0046 (2)	−0.0031 (2)
S2A	0.0159 (3)	0.0207 (3)	0.0252 (3)	−0.0071 (2)	0.0071 (3)	−0.0033 (2)
N1A	0.0121 (10)	0.0206 (10)	0.0135 (9)	−0.0053 (8)	0.0028 (8)	−0.0022 (8)
N2A	0.0129 (10)	0.0169 (10)	0.0190 (10)	−0.0034 (8)	0.0034 (8)	−0.0042 (8)
N3A	0.0145 (11)	0.0188 (10)	0.0180 (10)	−0.0069 (8)	0.0055 (9)	−0.0011 (8)
C1A	0.0115 (11)	0.0185 (11)	0.0149 (11)	−0.0025 (9)	−0.0003 (9)	−0.0044 (9)
C2A	0.0169 (12)	0.0198 (12)	0.0175 (12)	−0.0041 (10)	0.0010 (10)	−0.0034 (9)
C3A	0.0202 (13)	0.0192 (11)	0.0146 (11)	−0.0078 (10)	−0.0017 (10)	−0.0026 (9)
C4A	0.0294 (15)	0.0218 (12)	0.0206 (13)	−0.0090 (11)	0.0038 (11)	−0.0015 (10)
C5A	0.0333 (15)	0.0294 (14)	0.0180 (12)	−0.0185 (12)	0.0049 (11)	−0.0028 (10)
C6A	0.0246 (14)	0.0380 (15)	0.0162 (12)	−0.0153 (12)	0.0069 (11)	−0.0120 (11)
C7A	0.0215 (13)	0.0277 (13)	0.0209 (13)	−0.0097 (11)	0.0038 (11)	−0.0088 (10)
C8A	0.0205 (13)	0.0189 (11)	0.0123 (11)	−0.0095 (10)	0.0015 (10)	−0.0050 (9)
C9A	0.0134 (12)	0.0183 (11)	0.0124 (11)	−0.0042 (9)	−0.0022 (9)	−0.0014 (9)
C10A	0.0183 (13)	0.0209 (12)	0.0220 (13)	−0.0058 (10)	0.0054 (10)	−0.0045 (10)
C11A	0.0163 (12)	0.0186 (11)	0.0120 (11)	−0.0052 (9)	−0.0026 (9)	−0.0026 (9)
C12A	0.0212 (13)	0.0154 (11)	0.0196 (12)	−0.0039 (10)	0.0024 (10)	0.0005 (9)
C13A	0.0211 (13)	0.0227 (13)	0.0300 (14)	−0.0013 (10)	0.0028 (11)	−0.0050 (11)
S1B	0.0211 (3)	0.0214 (3)	0.0196 (3)	−0.0081 (2)	0.0061 (3)	−0.0087 (2)
S2B	0.0208 (3)	0.0305 (3)	0.0218 (3)	−0.0125 (3)	0.0100 (3)	−0.0099 (3)
N1B	0.0152 (10)	0.0216 (10)	0.0163 (10)	−0.0047 (8)	0.0034 (8)	−0.0017 (8)
N2B	0.0176 (11)	0.0252 (11)	0.0170 (10)	−0.0100 (9)	0.0050 (9)	−0.0074 (9)
N3B	0.0204 (12)	0.0247 (11)	0.0203 (11)	−0.0109 (9)	0.0112 (9)	−0.0084 (9)
C1B	0.0125 (12)	0.0153 (11)	0.0186 (12)	−0.0003 (9)	−0.0018 (10)	−0.0036 (9)
C2B	0.0152 (12)	0.0193 (11)	0.0145 (11)	−0.0038 (9)	0.0002 (9)	−0.0039 (9)
C3B	0.0130 (12)	0.0168 (11)	0.0157 (11)	0.0002 (9)	−0.0010 (9)	−0.0008 (9)
C4B	0.0204 (13)	0.0255 (13)	0.0145 (12)	−0.0033 (10)	0.0027 (10)	−0.0027 (10)
C5B	0.0191 (13)	0.0280 (13)	0.0196 (12)	0.0006 (10)	0.0041 (11)	0.0009 (10)
C6B	0.0183 (13)	0.0212 (12)	0.0315 (14)	−0.0020 (10)	0.0062 (11)	0.0048 (11)
C7B	0.0214 (13)	0.0211 (12)	0.0289 (14)	−0.0070 (10)	0.0051 (11)	−0.0059 (10)
C8B	0.0143 (12)	0.0177 (11)	0.0183 (12)	0.0004 (9)	0.0035 (10)	−0.0007 (9)
C9B	0.0126 (12)	0.0195 (11)	0.0136 (11)	−0.0013 (9)	−0.0020 (9)	−0.0033 (9)
C10B	0.0199 (13)	0.0264 (13)	0.0195 (12)	−0.0118 (10)	0.0066 (10)	−0.0083 (10)
C11B	0.0130 (12)	0.0200 (12)	0.0149 (11)	−0.0018 (9)	0.0011 (9)	−0.0037 (9)
C12B	0.0458 (18)	0.0446 (17)	0.0388 (16)	−0.0330 (14)	0.0295 (14)	−0.0292 (14)
C131	0.038 (3)	0.025 (3)	0.028 (3)	−0.011 (2)	0.005 (2)	−0.015 (2)
C132	0.027 (3)	0.029 (3)	0.021 (3)	−0.001 (2)	0.006 (2)	−0.004 (2)

### Geometric parameters (Å, °)

**Table:**

S1A—C8A	1.741 (2)	N1B—N2B	1.365 (3)
S1A—C1A	1.754 (2)	N2B—C11B	1.365 (3)
S2A—C11A	1.683 (2)	N2B—H2NB	0.852 (10)
N1A—C9A	1.291 (3)	N3B—C11B	1.334 (3)
N1A—N2A	1.375 (3)	N3B—C12B	1.453 (3)
N2A—C11A	1.360 (3)	N3B—H3NB	0.835 (10)
N2A—H2NA	0.852 (10)	C1B—C2B	1.369 (3)
N3A—C11A	1.337 (3)	C1B—C9B	1.449 (3)
N3A—C12A	1.467 (3)	C2B—C3B	1.429 (3)
N3A—H3NA	0.839 (10)	C2B—H2B	0.95
C1A—C2A	1.363 (3)	C3B—C4B	1.406 (3)
C1A—C9A	1.459 (3)	C3B—C8B	1.411 (3)
C2A—C3A	1.434 (3)	C4B—C5B	1.384 (3)
C2A—H2A	0.95	C4B—H4B	0.95
C3A—C4A	1.402 (3)	C5B—C6B	1.400 (4)
C3A—C8A	1.411 (3)	C5B—H5B	0.95
C4A—C5A	1.380 (3)	C6B—C7B	1.386 (3)
C4A—H4A	0.95	C6B—H6B	0.95
C5A—C6A	1.399 (3)	C7B—C8B	1.387 (3)
C5A—H5A	0.95	C7B—H7B	0.95
C6A—C7A	1.381 (3)	C9B—C10B	1.493 (3)
C6A—H6A	0.95	C10B—H10D	0.98
C7A—C8A	1.393 (3)	C10B—H10E	0.98
C7A—H7A	0.95	C10B—H10F	0.98
C9A—C10A	1.502 (3)	C12B—C132	1.432 (4)
C10A—H10A	0.98	C12B—C131	1.535 (4)
C10A—H10B	0.98	C12B—H12C	0.99
C10A—H10C	0.98	C12B—H12D	0.99
C12A—C13A	1.511 (3)	C12B—H12E	0.96
C12A—H12A	0.99	C12B—H12F	0.96
C12A—H12B	0.99	C131—H12F	1.13
C13A—H13A	0.98	C131—H13D	0.98
C13A—H13B	0.98	C131—H13E	0.98
C13A—H13C	0.98	C131—H13F	0.98
S1B—C8B	1.745 (2)	C132—H13G	0.98
S1B—C1B	1.750 (2)	C132—H13H	0.98
S2B—C11B	1.683 (2)	C132—H13I	0.98
N1B—C9B	1.298 (3)		
			
C8A—S1A—C1A	91.23 (11)	C9B—C1B—S1B	120.17 (17)
C9A—N1A—N2A	118.26 (18)	C1B—C2B—C3B	113.6 (2)
C11A—N2A—N1A	118.93 (18)	C1B—C2B—H2B	123.2
C11A—N2A—H2NA	120.8 (18)	C3B—C2B—H2B	123.2
N1A—N2A—H2NA	119.2 (18)	C4B—C3B—C8B	118.8 (2)
C11A—N3A—C12A	123.92 (19)	C4B—C3B—C2B	129.2 (2)
C11A—N3A—H3NA	117.9 (17)	C8B—C3B—C2B	111.9 (2)
C12A—N3A—H3NA	117.6 (17)	C5B—C4B—C3B	119.5 (2)
C2A—C1A—C9A	128.8 (2)	C5B—C4B—H4B	120.3
C2A—C1A—S1A	112.27 (17)	C3B—C4B—H4B	120.3
C9A—C1A—S1A	118.84 (16)	C4B—C5B—C6B	120.9 (2)
C1A—C2A—C3A	113.2 (2)	C4B—C5B—H5B	119.6
C1A—C2A—H2A	123.4	C6B—C5B—H5B	119.6
C3A—C2A—H2A	123.4	C7B—C6B—C5B	120.5 (2)
C4A—C3A—C8A	118.6 (2)	C7B—C6B—H6B	119.8
C4A—C3A—C2A	129.4 (2)	C5B—C6B—H6B	119.8
C8A—C3A—C2A	111.9 (2)	C6B—C7B—C8B	118.9 (2)
C5A—C4A—C3A	119.9 (2)	C6B—C7B—H7B	120.6
C5A—C4A—H4A	120.1	C8B—C7B—H7B	120.6
C3A—C4A—H4A	120.1	C7B—C8B—C3B	121.5 (2)
C4A—C5A—C6A	120.9 (2)	C7B—C8B—S1B	127.42 (19)
C4A—C5A—H5A	119.5	C3B—C8B—S1B	111.11 (17)
C6A—C5A—H5A	119.5	N1B—C9B—C1B	115.7 (2)
C7A—C6A—C5A	120.2 (2)	N1B—C9B—C10B	124.4 (2)
C7A—C6A—H6A	119.9	C1B—C9B—C10B	119.94 (19)
C5A—C6A—H6A	119.9	C9B—C10B—H10D	109.5
C6A—C7A—C8A	119.2 (2)	C9B—C10B—H10E	109.5
C6A—C7A—H7A	120.4	H10D—C10B—H10E	109.5
C8A—C7A—H7A	120.4	C9B—C10B—H10F	109.5
C7A—C8A—C3A	121.2 (2)	H10D—C10B—H10F	109.5
C7A—C8A—S1A	127.48 (18)	H10E—C10B—H10F	109.5
C3A—C8A—S1A	111.36 (17)	N3B—C11B—N2B	116.2 (2)
N1A—C9A—C1A	115.43 (19)	N3B—C11B—S2B	124.21 (18)
N1A—C9A—C10A	125.1 (2)	N2B—C11B—S2B	119.61 (18)
C1A—C9A—C10A	119.50 (19)	C132—C12B—N3B	122.0 (3)
C9A—C10A—H10A	109.5	C132—C12B—C131	68.4 (3)
C9A—C10A—H10B	109.5	N3B—C12B—C131	106.5 (3)
H10A—C10A—H10B	109.5	N3B—C12B—H12C	110.4
C9A—C10A—H10C	109.5	C131—C12B—H12C	110.4
H10A—C10A—H10C	109.5	C132—C12B—H12D	125.7
H10B—C10A—H10C	109.5	N3B—C12B—H12D	110.4
N3A—C11A—N2A	116.3 (2)	C131—C12B—H12D	110.4
N3A—C11A—S2A	123.46 (17)	H12C—C12B—H12D	108.6
N2A—C11A—S2A	120.25 (17)	C132—C12B—H12E	106.6
N3A—C12A—C13A	111.06 (19)	N3B—C12B—H12E	106.2
N3A—C12A—H12A	109.4	C131—C12B—H12E	143.4
C13A—C12A—H12A	109.4	H12C—C12B—H12E	72.4
N3A—C12A—H12B	109.4	C132—C12B—H12F	107.0
C13A—C12A—H12B	109.4	N3B—C12B—H12F	107.2
H12A—C12A—H12B	108.0	C131—C12B—H12F	47.0
C12A—C13A—H13A	109.5	H12C—C12B—H12F	140.9
C12A—C13A—H13B	109.5	H12D—C12B—H12F	66.4
H13A—C13A—H13B	109.5	H12E—C12B—H12F	106.9
C12A—C13A—H13C	109.5	C12B—C131—H13D	109.5
H13A—C13A—H13C	109.5	H12F—C131—H13D	76.5
H13B—C13A—H13C	109.5	C12B—C131—H13E	109.5
C8B—S1B—C1B	91.60 (11)	H12F—C131—H13E	141.6
C9B—N1B—N2B	117.9 (2)	C12B—C131—H13F	109.5
C11B—N2B—N1B	119.3 (2)	H12F—C131—H13F	103.3
C11B—N2B—H2NB	118.7 (18)	C12B—C132—H13G	109.5
N1B—N2B—H2NB	121.0 (18)	C12B—C132—H13H	109.5
C11B—N3B—C12B	124.4 (2)	H13G—C132—H13H	109.5
C11B—N3B—H3NB	117.9 (17)	C12B—C132—H13I	109.5
C12B—N3B—H3NB	116.7 (17)	H13G—C132—H13I	109.5
C2B—C1B—C9B	128.1 (2)	H13H—C132—H13I	109.5
C2B—C1B—S1B	111.75 (18)		
			
C9A—N1A—N2A—C11A	174.7 (2)	C8B—S1B—C1B—C2B	0.07 (17)
C8A—S1A—C1A—C2A	−0.36 (18)	C8B—S1B—C1B—C9B	178.56 (17)
C8A—S1A—C1A—C9A	177.19 (18)	C9B—C1B—C2B—C3B	−178.3 (2)
C9A—C1A—C2A—C3A	−176.5 (2)	S1B—C1B—C2B—C3B	0.1 (2)
S1A—C1A—C2A—C3A	0.8 (3)	C1B—C2B—C3B—C4B	−179.9 (2)
C1A—C2A—C3A—C4A	177.4 (2)	C1B—C2B—C3B—C8B	−0.2 (3)
C1A—C2A—C3A—C8A	−0.9 (3)	C8B—C3B—C4B—C5B	−0.7 (3)
C8A—C3A—C4A—C5A	0.1 (4)	C2B—C3B—C4B—C5B	179.0 (2)
C2A—C3A—C4A—C5A	−178.1 (2)	C3B—C4B—C5B—C6B	0.9 (3)
C3A—C4A—C5A—C6A	−1.0 (4)	C4B—C5B—C6B—C7B	−0.6 (3)
C4A—C5A—C6A—C7A	0.5 (4)	C5B—C6B—C7B—C8B	0.3 (3)
C5A—C6A—C7A—C8A	0.9 (4)	C6B—C7B—C8B—C3B	−0.2 (3)
C6A—C7A—C8A—C3A	−1.8 (4)	C6B—C7B—C8B—S1B	−179.72 (17)
C6A—C7A—C8A—S1A	177.30 (18)	C4B—C3B—C8B—C7B	0.4 (3)
C4A—C3A—C8A—C7A	1.3 (3)	C2B—C3B—C8B—C7B	−179.4 (2)
C2A—C3A—C8A—C7A	179.8 (2)	C4B—C3B—C8B—S1B	−179.99 (16)
C4A—C3A—C8A—S1A	−177.92 (18)	C2B—C3B—C8B—S1B	0.2 (2)
C2A—C3A—C8A—S1A	0.6 (3)	C1B—S1B—C8B—C7B	179.4 (2)
C1A—S1A—C8A—C7A	−179.3 (2)	C1B—S1B—C8B—C3B	−0.18 (17)
C1A—S1A—C8A—C3A	−0.14 (18)	N2B—N1B—C9B—C1B	−178.00 (18)
N2A—N1A—C9A—C1A	−178.12 (18)	N2B—N1B—C9B—C10B	1.4 (3)
N2A—N1A—C9A—C10A	1.3 (3)	C2B—C1B—C9B—N1B	172.1 (2)
C2A—C1A—C9A—N1A	173.7 (2)	S1B—C1B—C9B—N1B	−6.1 (3)
S1A—C1A—C9A—N1A	−3.4 (3)	C2B—C1B—C9B—C10B	−7.3 (3)
C2A—C1A—C9A—C10A	−5.8 (4)	S1B—C1B—C9B—C10B	174.51 (16)
S1A—C1A—C9A—C10A	177.12 (16)	C12B—N3B—C11B—N2B	174.0 (2)
C12A—N3A—C11A—N2A	−179.3 (2)	C12B—N3B—C11B—S2B	−5.2 (3)
C12A—N3A—C11A—S2A	0.3 (3)	N1B—N2B—C11B—N3B	7.4 (3)
N1A—N2A—C11A—N3A	8.8 (3)	N1B—N2B—C11B—S2B	−173.36 (15)
N1A—N2A—C11A—S2A	−170.77 (15)	C11B—N3B—C12B—C132	84.2 (4)
C11A—N3A—C12A—C13A	168.7 (2)	C11B—N3B—C12B—C131	158.7 (3)
C9B—N1B—N2B—C11B	177.9 (2)		

### Hydrogen-bond geometry (Å, °)

**Table:**

*D*—H···*A*	*D*—H	H···*A*	*D*···*A*	*D*—H···*A*
N3A—H3NA···N1A	0.84 (1)	2.27 (2)	2.623 (3)	106 (2)
N3B—H3NB···N1B	0.83 (1)	2.26 (2)	2.627 (3)	107 (2)
C10A—H10A···S2A^i^	0.98	2.84	3.374 (2)	115
N2A—H2NA···S2A^i^	0.85 (1)	2.81 (1)	3.638 (2)	164 (2)
C10B—H10D···S2B^ii^	0.98	2.82	3.373 (2)	117
C10A—H10B···Cg1^iii^	0.98	2.71	3.577 (2)	147
C10B—H10E···Cg2^iv^	0.98	2.72	3.600 (2)	150

Symmetry codes: (i) −*x*, −*y*, −*z*+2; (ii) −*x*+3, −*y*+2, −*z*+1; (iii) *x*+1, *y*, *z*; (iv) *x*−1, *y*, *z*.
